# COVID-19 reinfection in the presence of neutralizing antibodies

**DOI:** 10.1093/nsr/nwab006

**Published:** 2021-01-11

**Authors:** Ju Zhang, Nan Ding, Lili Ren, Rui Song, Danying Chen, Xuesen Zhao, Budong Chen, Junyan Han, Jiarui Li, Yangzi Song, Lin Di, Kai Han, Fengting Yu, Ruming Xie, Zhihai Chen, Wen Xie, Jingyuan Liu, Shan Cen, Yuhai Bi, Angela R Wu, Fujie Zhang, Chen Chen, Hui Zeng

**Affiliations:** Beijing Ditan Hospital, Capital Medical University, Beijing 100102, China; Beijing Key Laboratory of Emerging Infectious Diseases, Beijing 100102, China; Beijing Ditan Hospital, Capital Medical University, Beijing 100102, China; Beijing Key Laboratory of Emerging Infectious Diseases, Beijing 100102, China; NHC Key Laboratory of Systems Biology of Pathogens and Christophe Mérieux Laboratory, Institute of Pathogen Biology, Chinese Academy of Medical Sciences & Peking Union Medical College, Beijing 100730, China; Key Laboratory of Respiratory Disease Pathogenomics, Chinese Academy of Medical Sciences and Peking Union Medical College, Beijing, 100730, China; Beijing Ditan Hospital, Capital Medical University, Beijing 100102, China; Beijing Ditan Hospital, Capital Medical University, Beijing 100102, China; Beijing Key Laboratory of Emerging Infectious Diseases, Beijing 100102, China; Beijing Ditan Hospital, Capital Medical University, Beijing 100102, China; Beijing Key Laboratory of Emerging Infectious Diseases, Beijing 100102, China; Beijing Ditan Hospital, Capital Medical University, Beijing 100102, China; Beijing Ditan Hospital, Capital Medical University, Beijing 100102, China; Beijing Key Laboratory of Emerging Infectious Diseases, Beijing 100102, China; Beijing Ditan Hospital, Capital Medical University, Beijing 100102, China; Beijing Key Laboratory of Emerging Infectious Diseases, Beijing 100102, China; Beijing Ditan Hospital, Capital Medical University, Beijing 100102, China; Beijing Key Laboratory of Emerging Infectious Diseases, Beijing 100102, China; Beijing Advanced Innovation Center for Genomics, Biomedical Pioneering Innovation Center, Peking University, Beijing 100871, China; Beijing Ditan Hospital, Capital Medical University, Beijing 100102, China; Beijing Key Laboratory of Emerging Infectious Diseases, Beijing 100102, China; Beijing Ditan Hospital, Capital Medical University, Beijing 100102, China; Beijing Ditan Hospital, Capital Medical University, Beijing 100102, China; Beijing Ditan Hospital, Capital Medical University, Beijing 100102, China; Beijing Ditan Hospital, Capital Medical University, Beijing 100102, China; Beijing Ditan Hospital, Capital Medical University, Beijing 100102, China; Institute of Medicinal Biotechnology, Chinese Academy of Medical Sciences and Peking Union Medical School, Beijing 100050, China; CAS Key Laboratory of Pathogenic Microbiology and Immunology, Institute of Microbiology, Center for Influenza Research and Early-warning (CASCIRE), CAS-TWAS Center of Excellence for Emerging Infectious Diseases (CEEID), Chinese Academy of Sciences, Beijing 100101, China; University of Chinese Academy of Sciences, Beijing 101408, China; Division of Life Science and Department of Chemical and Biological Engineering, Hong Kong University of Science and Technology, Hong Kong SAR, China; Beijing Ditan Hospital, Capital Medical University, Beijing 100102, China; Beijing Ditan Hospital, Capital Medical University, Beijing 100102, China; Beijing Key Laboratory of Emerging Infectious Diseases, Beijing 100102, China; Beijing Ditan Hospital, Capital Medical University, Beijing 100102, China; Beijing Key Laboratory of Emerging Infectious Diseases, Beijing 100102, China

**Keywords:** COVID-19, SARS-CoV-2, reinfection, neutralizing antibody

## Abstract

In the face of the coronavirus disease 2019 (COVID-19), strong and long-lasting immunity is required to protect the host from secondary infections. Recent studies revealed potential inadequacy of antibodies against SARS-CoV-2 in some convalescent patients, raising serious concerns about COVID-19 reinfection. Here, from 273 COVID-19 patients, we identified six reinfections based on clinical, phylogenetic, virological, serological, and epidemiological data.

During the second episode, we observed re-emergence of COVID-19 symptoms, new pulmonary lesions on CT images, increased viral load, and secondary humoral immune responses. The interval between the two episodes ranged from 19 to 57 days, indicating COVID-19 reinfections could occur after a short recovery period in convalescent patients. More importantly, reinfection occurred not only in patients with inadequate immunity after the primary infection, but also in patients with measurable levels of neutralizing antibodies. This information will aid the implementation of appropriate public health and social measures to control COVID-19, as well as inform vaccine development.

ORIGINAL UNEDITED MANUSCRIPT

## INTRODUCTION

Due to the high transmissibility of the SARS-CoV-2 virus, it continues to infect over 300,000 people worldwide per day (WHO statistics, December, 2020), even almost one year after the first COVID-19 cases were confirmed [1, 2]. One of the most important public health discussions being undertaken currently is concerning the protective nature of the immune response and the possibility of reinfection in recovered individuals. Since seroconversion of SARS-CoV-2 antibodies has been detected in convalescent COVID-19 patients [3], many governments and public health agencies have proposed the idea of an "immunity passport" to help with recovery of community social and economic norms: recovered individuals or those with detectable levels of antibodies against SARS-CoV-2 issued with an immunity passport can be less stringent with lockdown, social distancing, or travel rules, which would enable these individuals to travel or return to work [4, 5].

However, a number of clinical studies have also revealed low titers of antibodies or rapid waning of antibodies against SARS-CoV-2 in convalescent patients [3, 6], and raised concerns regarding the risks of SARS-CoV-2 reinfection. There have been anecdotal cases independently reported by several groups of patients testing positive for SARS-CoV-2 virus again after recovery, appearing to be recurrence of the infection [7, 8]; several case studies of reinfection with a second bout of SARS-CoV-2 have also been identified in several countries [9-13]. However, more evidence is needed to distinguish *bona fide* reinfection of live replicating virus from both false positive PCR results due to residual viral RNA, as well as

ORIGINAL UNEDITED MANUSCRIPT

from a recurrence of primary infection.

In our study of 273 patients, we report six cases of reinfection that all had negative PCR test results between the positive PCR tests during the two infection periods. In five of the six patients, viral genome sequencing results show unambiguous infection of a distinct viral strain in the second episode that was not in wide circulation prior to the time of secondary infection, ruling out the possibility of a relapse from primary infection. Of note, reinfection could occur shortly after recovery from primary infection. In addition, some of these patients mounted immune responses within the range that would be considered protective based on prior studies, yet were reinfected. These findings have strong and important implications for public health policy decisions, as well as in guiding efficacy assessment and development of vaccines.

## RESULTS

### Six COVID-19 reinfection cases confirmed by nucleic acid tests and viral genome sequencing

From January 29 to April 30 of 2020, Beijing Ditan Hospital admitted 273 cases of COVID-19, including 152 community-linked cases who were diagnosed from January 20$^{th}$ to March 9$^{th}$, 2020, and 121 international-linked cases (101 from Europe and 15 from North America) (detail in Supplementary Methods & Results 1.1) who were diagnosed from February 29$^{th}$ to April 17$^{th}$, 2020. The viral genome from two periods displayed a distinct pattern [14]. We monitored the clinical and laboratorial data during the hospitalization and

ORIGINAL UNEDITED MANUSCRIPT

followed up these patients after they discharged, when they met the recovery criteria according to National Health Commission of the People's Republic of China guidelines (no fever ≥ 2 days; obvious improvement of respiratory symptoms and pulmonary images; twice consecutive negative RT-PCR) [15]. From March 1st, we found 28 patients who have twice consecutive positive tests for SARS-CoV-2 during follow-up among these patients.

To determine which of these are true reinfections, we used the MINERVA sequencing strategy [16] on paired clinical specimens obtained in two episodes of these 28 patients. We ultimately obtained paired complete viral genomes from seven patients. Phylogenomic analysis on these complete viral genomes showed one of the paired-genome from the same lineage, while the other six paired-genomes were attributed to different lineages or descending lineages with 3-11 distinct SNPs between each pair (Table 1 & S1). A different viral lineage between the first and second infection episodes, particularly with the negative PCR results between episodes (Table S2), is strong evidence of true reinfection rather than false positive or relapse of the primary infection. More importantly, five of these six pairs sampled in the second episodes were found to be D614G mutants (Table 1). This variant was almost completely absent in China prior to March [14], and was identified as the predominant variant in Europe, gradually becoming frequent worldwide toward the end of March [17]. Furthermore, each of viral genomes sequenced contained a different set of SNPs at various positions (Table 1), which made it extremely unlikely that these were the result of cross contamination between patient samples; if a single or a few samples contaminated others, the SNPs observed would be the same across samples which was not the case here. To further validate sample identity,

ORIGINAL UNEDITED MANUSCRIPT

we additionally performed host mitochondrial DNA analysis to rule out the possibility of sample mix-ups (Fig. S1, Supplementary Methods & Results 1.3). Taken together, these data provide robust molecular evidence for reinfection from *bona fide* replicating virus in at least six patients.

**Epidemiological and clinical data of patients with reinfection**

Next, we assessed the clinical and epidemiological data of these six patients. As summarized in Table S2, they comprised five adults (age: 33-84 years) and one 2-year-old child. Two were critical COVID-19 patients (P1, P6), and the other four were moderate cases (P2-P5) in their primary infections. None of them have autoimmune diseases, cancer, or a history of immunosuppressive drug use. The interval between the end of the primary infection and the beginning of the reinfection ranged from 19 to 57 days (Fig. 1A, Table S2). All patients had at least two negative viral RNA tests during the recovered phase between infection periods; two of them (P1 and P2) had 12 and five negative viral RNA tests respectively during this recovered phase (Fig. 1A, Table S2). During the secondary infection, five cases (P1-P4, P6) tested positive for SARS-CoV-2 viral RNA 3-14 times, and four patients (P1-P3, P6) had low cycle threshold (Ct) values, ranging from 24 to 28, representing high viral loads. Viral RNA positive durations lasted at least nine days in five cases (P1-P4, P6; Fig. 1A, Table S2). No difference in the infection duration and the viral loads of the SARS-CoV-2 RNA was observed between the primary infections and secondary infections (Table S2). Three cases (P1-P3) developed symptoms again after the first negative intervals, including fever, cough, expectoration, and stuffy nose (Fig. 1A, Table S2). Meanwhile, CT scan of P1 and P2

ORIGINAL UNEDITED MANUSCRIPT

exhibited new infected lesions, including patchy ground-glass opacity and consolidation in the chest (Fig. 1B & S2). These clinical data further support the notion that these patients were reinfected new viruses rather than re-testing positive from the same primary infection.

We also performed contact-tracing to assess potential sources of reinfection. Among these patients, case P1 stayed in the intensive care unit due to concomitant cardiorenal complications after prior negative COVID-19 test. She had a history of sharing ward with other COVID-19 patients, and tested positive for viral RNA again during hospitalization. The other five cases (P2-P6) re-tested positive during the follow-up after discharge. Case P5 had a contact history with a confirmed COVID-19 patient with an identical viral genome except for one SNP difference before his second episode (Table S1). Case P2 revisited the hospital after developing COVID-19 symptoms. The other three patients (P3, P4, and P6) were self-quarantined for 14 days according to the Diagnosis and Treatment Protocol for COVID-19, and re-visited the hospital for follow-up and re-testing.

**Reinfection occurred in the presence of varied levels of neutralizing antibodies**

We further assessed the dynamics of antibodies response by measuring specific IgM/IgG targeting the RBD of S protein (RBD) or N protein (Fig. 1C & S3, Table S3). Various antibody levels were observed in convalescent serum/plasma in these six patients. P2 and P4 had low anti-RBD IgM/IgG (≤1:40) responses to the primary infection. In the other three patients (P1, P3 and P6), titers reached 1:80-1:320 for RBD-IgM and IgG (Fig. 1C, Table S3). During the secondary infection, cases P1 and P2 displayed secondary immune responses with

ORIGINAL UNEDITED MANUSCRIPT

an increase in serum antibody titers: for example, case P1 RBD-IgM increased from 1:10 to 1:2560, NP-IgM from 1:20 to 1:80, and NP-IgG from 1:640 to 1:2560 (Fig. 1C & S3, Table S3).

Since five of the six cases (P1-P5) were infected with the D614G variants in the secondary infection (Table 1), we also performed microneutralization assays with live virus of both reference SARS-CoV-2 and D614 variants in parallel. Consistent with ELISA data, the samples from case P2 and P4 exhibited low inhibitory dilution 50 (ID50) during the primary and secondary infections; whereas the samples from case P1 and P3 had a certain amount of neutralizing activity against both reference SARS-CoV-2 and D614 variants (Fig. 1C, Table S3). Of note, the neutralizing titers against live virus with D614G mutation were 1:18.6 (P1) and >1:64 (P3), respectively (Fig. 1C, Table S3). We compared these data with the previous study on 454 convalescent samples from 178 COVID-19 confirmed patients with the identical microneutralization system [18] (neutralizing titer: median, 1:19; QRI, 1:10-1:28.2; Fig. S4), and the ID50 of case P1 and P3 exceeded 44.3% and 97.4% samples of COVID-19 patients (detail in the Supplementary Methods & Results 1.8).

Generally, the antibody titer and neutralization ability were in agreement for all reinfection cases (Fig. 1C), consistent with the literature [18, 19]. In a prior SARS-CoV-2 studies, recovered patients with antibody titers above 1:160 can maintain stable serum antibody levels for up to 148 days [18, 19]. We observed three patients with initially higher antibody titers after recovery (P1, P3, P6) were able to maintain these levels well into the secondary

ORIGINAL UNEDITED MANUSCRIPT

infection (Fig. 1C). Furthermore, we observed the neutralizing titers against both SARS-CoV-2 reference strain and D614G variants increased drastically in sera/plasma of case P1 after secondary infection, suggesting that the immune memory response was activated after secondary challenge (Fig. 1C & S3, Table S3). But in all of these cases, P1, P3, and P6, reinfection occurred nonetheless, suggesting that potential COVID-19 reinfections could still occur even in individuals with measurable levels of neutralizing antibodies.

**Discussion**

Reinfection has been observed in seasonal beta-coronavirus, such as HKU1 and OC4316 [20]; recently single cases of SARS-CoV-2 reinfection were also reported in Hong Kong, China [9]; the United States [10], Ecuador [11], Belgium [12], and the Netherlands [13]. Herein, we identified six cases of reinfection. Importantly, the time interval between the two bouts of infection in the present study ranged from 19 to 57 days (Fig. 1A, Table S2), indicating that reinfection could occur much earlier than previously suspected.

The D614G haplotype was almost non-existent in China during the time of the primary infection, but was found as the variant in five of the six reinfections (Table 1). Given the timing of the introduction of this haplotype to China, there is overall compelling evidence proving that the viral variants in the secondary infection are different from that of the first, and that the main variants (D614G) found in the second infections are highly unlikely to have been involved in the first infections. In addition to viral phylogenetic data, the RT-PCR assays performed by a laboratory outside of the hospital as well as the clinical findings during the

ORIGINAL UNEDITED MANUSCRIPT

second episode collectively strengthen the assumption that these are *bona fide* SARS-CoV-2 reinfection: During the secondary infection 1) patients tested positive for SARS-CoV-2 RNA for 3-26 days; 2) the lowest Ct value of the SARS-CoV-2 RNA for each patient was 24-37 during this period; 3) COVID-19-related symptoms reappeared accompanied by new pulmonary inflammatory lesions; 4) microneutralization assays revealed secondary humoral immune responses (Figure 1, Table S2). Together, these are compelling evidences of the second episode being caused by a new virus, rather than a persistence of the virus causing the first episode.

Whether reinfection would occur in an individual is not only determined by the magnitude and duration of that individual's specific immunity, but also varied circumstances of their exposure risk to the virus. The conventional wisdom is that immunity would protect the recovered patients for a long period, and the possibility of reinfection was not considered or taken seriously. Of note, despite strict public health policies and social distancing measurements in Beijing, we still identified multiple cases of SARS-CoV-2 reinfection by monitoring "recovered" patients, suggesting that reinfection might not be a rare event as many previously thought. However, it is not feasible to speculate or derive the population rate or risk of refection based on these data. Considering the escalation of the pandemic in many countries around the world, as well as the continued insufficiency of diagnostic resources in many communities, the risk of re-exposure to SARS-CoV-2 is still high, and the reinfection certainly is a cause for alarm or concern.

ORIGINAL UNEDITED MANUSCRIPT

It has been proposed that reinfection is a result of inadequate humoral protection against SARS-CoV-2. Consistent with this notion, in the Hong Kong reinfection case the patient did not have adequate amounts of virus-specific antibodies [9]. This is also the case for some patients in this study. However, a recent study revealed that robust neutralizing antibodies to SARS-CoV-2 infection remain relatively stable for several months after infection [19, 21]. Concordant with their findings, we also note substantial titers of neutralizing antibodies before the secondary infection in several patients in this study (Fig. 1C, Table S3). This is further evidence that post-convalesce patients with the neutralizing antibodies should not be considered safe against a second bout of infection.

Substantial work has been done to investigate the possible changes in SARS-CoV-2 infectivity and immune evasion, and so far, there is little evidence to suggest viral evolution that trends toward increasing viral ability to re-infect or evade antibodies [22]. In this study, the viral genomes sequenced from the secondary infections are found to be of a distinct lineage from that of the primary infection (Table S1), as confirmed by phylogenetic classification algorithms [23], and genetic differences between samples were found with high confidence. Further comparisons of complete viral genomes between primary and secondary infections in each paired sample revealed 3-11 SNPs (Table 1), but interestingly, none of the genomic differences were in the RBD region. In addition, although five viral genomes from the second infection contained a common mutation D614G in the S protein (Table 1), serum from these patients was still able to neutralize D614G viral variants *in vitro* (Fig. 1C, Table S3) [22], negating the possibility that these mutations afford the virus with immune escape

ORIGINAL UNEDITED MANUSCRIPT

capabilities.

Clearly, there remains much unknown about COVID-19, and characterizing the underlying mechanism of reinfection could help to inspire development of new vaccines that are safer, more effective, and more protective. That SARS-CoV-2 can cause a second infection in the presence of measurable antibody responses calls into question whether a threshold level of antibody response is required for protection. Vaccine development will need to consider not just whether a response is raised, but also the quantitative level of antibody response raised as an important endpoint for testing and trials. Furthermore, vaccine-induced immunity might be different from natural immunity; vaccines may not induce the same T cell responses as a true SARS-CoV-2 infection does [24]. Studies have accumulated evidence of more broadly dysregulated immune function during viral infection [25], and consistently, we also observed decreased $CD4^+$ T cell counts in five adult cases, and decreased $CD8^+$ T cell counts in two critical COVID-19 patients (Table S4 & S5). Therefore, an effective vaccine may also need to mount T cell immunity, and development of vaccines that elicit both neutralizing antibodies and T-cell responses, which may be achievable by using a combination of different vaccine types and approaches, should be encouraged [26].

Summarily, we demonstrated that COVID-19 reinfections could occur during the convalescent stage, even in cases with natural infection induced humoral immunity. Several cases also developed characteristic COVID-19 symptoms. Both investigation of unique patient cohorts such as those in this study, as well as reasonable and responsible discussions

ORIGINAL UNEDITED MANUSCRIPT

about the results have important implications for public health. Specifically, to effectively counter the pandemic and aid individual patient recovery, we need both effective and protective vaccines in addition to policies that promote personal protective behaviors for as long as necessary. Inappropriate public health measures could result in recurring waves of COVID-19, thus, public health policies, vaccine development and assessment strategies should be carefully evaluated in light of this study's findings.

**Methods**

See supplemental appendix.

**Summary Points**

1) Using phylogenetic lineage determination system based on complete viral genomes, along with corresponding clinical data, we provide definitive evidences for six cases of COVID-19 reinfection in which recovery from the primary infection.

2) Reinfection could occur shortly after recovery from the primary infection. Reinfected patients may display COVID-19 symptoms, new infection-associated lesions in CT images and secondary humoral responses.

3) Reinfection of SARS-CoV-2 could occur not only in patients with inadequate or loss of immunity, but also in some patients who developed and maintained a high neutralizing antibody response.

ORIGINAL UNEDITED MANUSCRIPT

**Acknowledgements**

We thank all health care workers involved in the diagnosis and treatment of the COVID-19 in Beijing Ditan Hospital. This work was supported by National Key Research and Development Project of China (2020YFC 0861200), Beijing Municipal Science and Technology Project (Z201100007920017), Beijing Hospital Authority (Grant No. DFL20191801).

**Author contributions**

J.Z., N.D. and L.R. contributed to conceptualization, data collection, data analysis, results interpretation and manuscript writing. H.Z., F. Z. and A.W. contributed to conceptualization, results interpretation and manuscript writing. C.C. and R. S. contributed to data analysis and manuscript writing. D.C., X.Z., B.C., J.H., J.L., Y.S., L.D., K.H., F.Y., R.X., Z.C., W.X., J.L., S.C. and Y.B. contributed to data collection and results interpretation.

**Competing interests**

The authors declare no competing interests.

ORIGINAL UNEDITED MANUSCRIPT

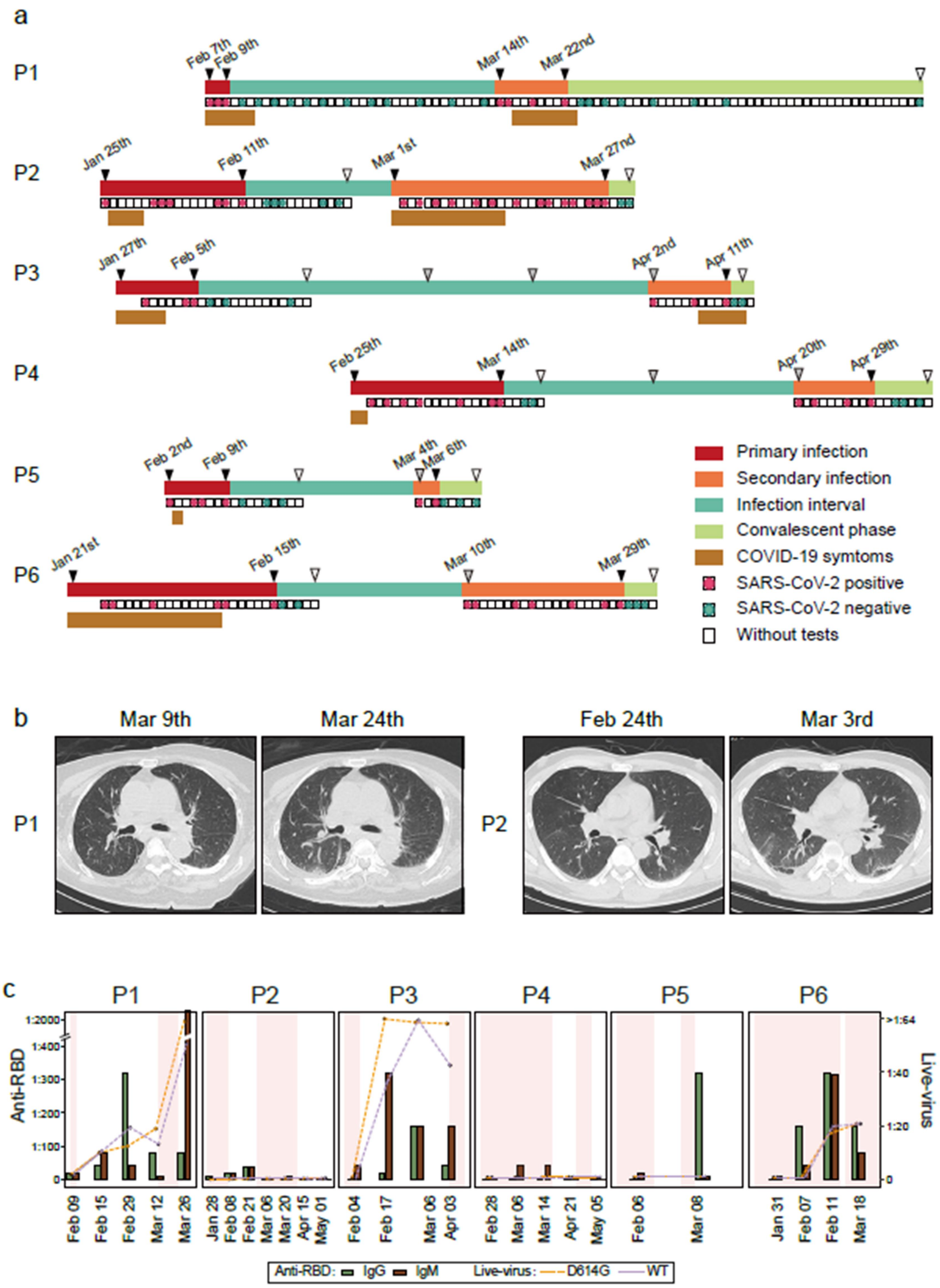

**Figure 1. Detailed timelines and high-resolution chest CT scans of reinfected cases. A**, Timeline of the six re-infected COVID-19 patients. Infection stages are highlighted in different colors on the first line for each case. RT-PCR results and duration of hospitalization are shown in the second lines for each patient. Durations of COVID-19 symptoms are shown in the third lines. Red line, primary infection; orange line, secondary infection; green line, infection interval between two infection episodes; light green line, convalescent phase of the secondary infection; red dot, positive RT-PCR tests; green dot, negative RT-PCR test; tan line, symptom duration; grey arrows, follow-up dates initialed by doctors or patients; white arrows, discharge dates from hospital. **B**, Representative high-resolution CT images before and after the secondary infections. Increasing and multifocal ground-glass changes in subpleural areas were observed (P1: dorsal segment of right lower lobe; P2: right upper lobe, left lingual lobe and bilateral lower lobes). **C**, Titer dynamics of antibodies against SARS-CoV-2 RBD, and neutralizing activity against live virus of reference SARS-CoV-2 and D614 variants in six patients. Green bars, anti-RBD IgG titers; sienna bars, anti-RBD IgM titers; line and dots, neutralizing ID50 against authentic virus of SARS-CoV-2 reference strain (purple) and D614G variant (orange). Background color shows the infection period, with the first and second infection periods shaded and recovered periods in white.

ORIGINAL UNEDITED MANUSCRIPT

**Table 1. SNPs on the viral genomes sequenced from the six reinfected patients**

| SNP | Position | Gene | Class | Amino Acid Substitution | Sample | | | | | | | | | | | |
|---|---|---|---|---|---|---|---|---|---|---|---|---|---|---|---|---|
| | | | | | **P1.1** | **P1.2** | **P2.1** | **P2.2** | **P3.1** | **P3.2** | **P4.1** | **P4.2** | **P5.1** | **P5.2** | **P6.1** | **P6.2** |
| C8782T | 8782 | *ORF1ab* | synonymous variant | p.2839S | C | C | C | C | C | C | C | C | C | C | **T** | C |
| T28144C | 28144 | *ORF8* | missense variant | p.84L>S | T | T | T | T | T | T | T | T | T | T | **C** | T |
| C241T | 241 | *5'UTR* | upstream gene variant | - | C | **T** | C | **T** | C | **T** | C | **T** | C | **T** | C | C |
| C14408T | 14408 | *ORF1ab* | missense variant | p.4715P>L | C | **T*** | C | **T** | C | **T** | C | **T*** | C | **T** | C | C |
| A23403G | 23403 | *S* | missense variant | p.614D>G | A | **G** | A | **G** | A | **G** | A | **G*** | A | **G** | A | A |
| C3037T | 3037 | *ORF1ab* | synonymous variant | p.924F | C | N | C | **T** | C | **T** | C | **T** | C | **T** | C | C |
| G26144T | 26144 | *ORF3a* | missense variant | p.251G>V | **T** | G | G | G | **T** | G | G | G | G | G | G | G |
| G3231T | 3231 | *ORF1ab* | missense variant | p.989G>V | G | **T** | G | G | G | G | G | G | G | G | G | G |
| T16254C | 16254 | *ORF1ab* | synonymous variant | p.5330V | T | **C** | T | T | T | **C** | T | T | T | T | T | T |
| T16545C | 16545 | *ORF1ab* | synonymous variant | p.5427V | T | **C** | T | T | T | T | T | T | T | T | T | T |
| A26449T | 26449 | *E* | stop gained | p.69R>* | A | **T** | A | A | A | A | A | A | A | A | A | A |
| C313T | 313 | *ORF1ab* | synonymous variant | p.16L | C | C | C | **T** | C | C | C | C | C | C | C | C |
| T14950C | 14950 | *ORF1ab* | missense variant | p.4896F>L | T | T | T | **C** | T | T | T | T | T | T | T | T |
| G28881A | 28881 | *N* | missense variant | p.203R>K | G | N | G | **A** | G | G | G | G | G | G | G | G |
| G28882A | 28882 | *N* | synonymous variant | p.203R | G | N | G | **A** | G | G | G | G | G | G | G | G |
| G28883C | 28883 | *N* | missense variant | p.204G>R | G | N | G | **C** | G | G | G | G | G | G | G | G |
| G13617T | 13617 | *ORF1ab* | missense variant | p.4451K>N | G | G | G | G | **T** | G | G | G | G | G | G | G |
| C337T | 337 | *ORF1ab* | synonymous variant | p.24R | C | C | C | C | C | C | **T** | C | C | C | C | C |
| C3429T | 3429 | *ORF1ab* | missense variant | p.1055T>I | C | N | C | C | C | C | **T** | C | C | C | C | C |
| C6268T | 6268 | *ORF1ab* | synonymous variant | p.2001A | C | C | C | C | C | C | **T** | C | C | C | C | C |
| C18512T | 18512 | *ORF1ab* | missense variant | p.6083P>L | C | C | C | C | C | C | **T** | C | C | C | C | C |
| G237T | 237 | *5'UTR* | upstream gene variant | - | G | G | G | G | G | G | G | **T** | G | G | G | G |
| G2229A | 2229 | *ORF1ab* | missense variant | p.655C>Y | G | G | G | G | G | G | G | **A** | G | G | G | G |
| T19561A | 19561 | *ORF1ab* | missense variant | p.6433L>M | T | T | T | T | T | T | T | **A** | T | T | T | T |
| T4008C | 4008 | *ORF1ab* | missense variant | p.1248F>S | T | T | T | T | T | T | T | T | **C** | T | T | T |
| T28031C | 28031 | *ORF8* | synonymous variant | p.46Y | T | T | T | T | T | T | T | T | **C** | T | T | T |
| C1059T | 1059 | *ORF1ab* | missense variant | p.265T>I | C | C | C | C | C | C | C | N | C | **T** | C | C |

ORIGINAL U

| | | | | | | | | | | | | | | | | |
|---|---|---|---|---|---|---|---|---|---|---|---|---|---|---|---|---|
| G25217T | 25217 | *S* | missense variant | p.1219G>C | G | G | G | G | G | G | G | G | G | **T** | G | G |
| G25563T | 25563 | *ORF3a* | missense variant | p.57Q>H | G | G | G | G | G | G | G | G | G | **T** | G | G |
| C27389T | 27389 | *Intergenic* | downstream gene variant | - | C | C | C | C | C | C | C | C | C | C | C | **T** |

* shows the SNPs validated by PCR-Sanger sequencing approach, which were not covered by enough reads after removing duplicates.   N=A or C or G or T.

ORIGINAL UNEDITED MANUSCRIPT
