# Supplementary material for: COVID-19 reinfection in the presence of neutralizing antibodies
Source: Natl Sci Rev. 2021 Jan 11;8(4):nwab006. doi: 10.1093/nsr/nwab006 (PMC7928639; doi:10.1093/nsr/nwab006)
Supplement: nwab006_Supplemental_File [file nwab006_supplemental_file.docx]

**­** **Supplemental Appendix**

**Supplemental** **Methods & Results**

**1.1 Cases.** Our hospital has received a total of 273 cases from Jan 20 to Apr 30, 2020, accounting for 46.04% Beijing cases (593 cases) in the same period. The 273 individuals consisted of 136 (~49.8%) females and 137 (~50.2%) males, with a median age of 38.7 years (Range: 0.6-93; QRI: 23-52). 97 of them were Beijing local cases (no travel history associated with Wuhan), 55 of them with Wuhan-contact history, and 121 of them with travel history from other countries around the world [1]. This study was approved by the Ethics Committee of Beijing Ditan Hospital, Capital Medical University (No. JDLK2020-042-02).

All the patients were diagnosed and treated based on the guidelines for the diagnosis and treatment of COVID-19 from the National Health Commission of the People's Republic of China [2]. We recorded and analyzed their clinical symptoms, physical findings, and hematological, biochemical, radiological, and microbiological data. Samples from pharyngeal swabs, sputum, feces and blood were collected according to the clinical guidelines, encoded with individual serial numbers, and compiled into traceable electronic files.

The cases of COVID-19 reinfection were defined according to the following criteria: 1) recurrent COVID-19 cases, ever meeting the recovery criteria with at least two consecutive SARS-CoV-2 negative tests; 2) recurrence confirmed by at least two positive tests after recovery; 3) the interval between two infections >14 days; 4) paired viral genomes from either the primary or the secondary infection were assigned to different phylogenetic lineages. The infection duration was calculated from symptom onset or the laboratory confirmation to SARS-CoV-2 test turning negative. The interval between two infection stages was calculated from the last SARS-CoV-2 positive test of the primary course to the first positive test of the secondary course.

The contact tracing of the patients was performed by reviewing the medical records of each patient and follow-up by phone. The detail tracing information of the six cases have been listed in the supplementary appendix.

**1.2 RNA extraction and RT-PCR for SARS-CoV-2 detection.** Viral RNA was extracted from clinical specimens using the QIAamp® Viral RNA Mini Kit according to the manufacturer’s instructions, and was determined by RT-PCR targeting the open reading frame 1ab (*ORF1ab*) gene and nucleoprotein (*N*) gene of SARS-CoV-2 as previously described (Shanghai BioGerm Medical Technology Co. Ltd., China; DaAn Gene Co. Ltd., China). When Ct values of the both targets were less than or equal to 38, the RT-PCR test was interpreted as positive for SARS-CoV-2, according to Chinese national guidelines. All the positive tests were independently confirmed by Beijing Ditan Hospital and Chinese Center for Disease Control and Prevention.

**1.3 Rule out the possibility of sample mix-up.** To exclude the possibility of sample mix-up, we used the paired meta-transcriptome sequencing data (Table S2) of the six re-infected cases to perform the host mitochondrial DNA (mtDNA) haplotype analysis, which could assess genetic relationships of individuals within a species and be adopted in forensics [3-5]. We mapped the clean reads to GRCh38 genome (Gencode v31) using STAR aligner [6] after trimming adaptors and low-quality bases with cutadapt [7]. Unique alignment of mitochondrion was used to call SNPs using mpileup tool in samtools and customized Perl script. Confident SNPs with variant allele frequency ≥ 0.5, and site depth > 0 in paired data (omitting mutual SNPs of the six cases) were chosen to draw clustering heatmap (Fig. S1). It showed that the paired sequencing data from the same re-infected case were assigned to the same mtDNA haplotype, indicating no sample mix-ups during the procedures of sample collection and management (Fig. S1). In addition, we confirmed the result with identical genome sequence in one patient (P3, Table S2).

**1.4 Complete genome sequencing for the viral genome.** We used the SHERRY based sequencing strategy to obtain the complete genome sequences of SARS-CoV-2 [2, 8]. Briefly, we performed rRNA removal using the MGIEasy rRNA Depletion Kit (Beijing genomics institute Co. Ltd., China), and then performed reverse transcription with 200 pmole of random decamer and 20 pmole of T30VN primer. The cDNA/RNA hybrid was then tagmented with Tn5 transposome (Vazyme Biotech Co. Ltd., China). After gap filling and PCR, the meta-transcriptome libraries underwent further SARS-CoV-2 sequence enrichment using biotinylated RNA probes targeting the whole viral genome of SARS-CoV-2 (iGeneTech, China). The final viral-enriched libraries were sequenced on an Illumina NextSeq500 platform in 2x75bp pair-end mode. The approach generated 21.5 million (QRI: 10.5-56.9 million) viral reads per sample, respectively (Table S2).

**1.5 Viral genome analysis.** Sequencing raw reads were subjected to quality control procedure using BBmap (version 38.68) before further analysis. Adaptors and low-quality bases (base quality < 20) were trimmed, and reads <30 length were discarded with Cutadapt (version 1.15) [7]. Qualified reads were mapped to the reference SARS-CoV-2 genome (Wuhan-Hu-1, GenBank accession MN908947) using Bowtie2 (version 2.2.5) with default parameters [9, 10]. Duplicate reads were removed from the primary alignment with Picard Tools (version 1.141) (http://broadinstitute.github.io/picard). We used mpileup in samtools (version 1.10) to call SNPs with parameter -Q 20 [11]. We called single nucleotide variants if the depth ≥5 and major allele frequency >75%, and discarding the sites only supported by a single strand. Consensus sequences were called using BCFtools based on reference sequence. Phylogenetic robust lineage was evaluated using pangolin as described previously [12].

**1.6 Detection of the SARS-CoV-2 specific IgG and IgM antibody.** Total 26 sequential serum/plasma samples were collected from the six patients. Using indirect ELISA (enzyme linked immunosorbent assay) kits (Beijing Hotgen and Guangzhou Qianxun Biotechnology Co. Ltd., China) according to its manual, anti-SARS-CoV-2 antibody (IgM and IgG) titers were assessed against the RBD region of spike protein and the NP protein. Briefly, 100 ng recombinant RBD or NP proteins were coated in 96-well plates and stored at 4℃. Serum/plasma samples were inactivated at 56℃ for 30 minutes, and a serial of two-fold dilutions were performed for the antibody tests. HRP (horseradish peroxidase) conjugated secondary antibody against human IgG/IgM were then incubated in the reaction system, and the spectrophotometric signals were measured at 450nm in microplate reader. Three duplicate tests were performed for each antibody dilution.

**1.7 Microneutralization Assay.** The microneutralization assay was performed in a certified Biosafety Level 3 lab. The serial two-fold dilution of plasma from 1:4 was preincubated with reference SARS-CoV-2 (IPBCAMS-WH-V03) or D614G variant (IPBCAMS_BJ95) live virus at 100 TCID50 determined by using Vero cells (CCL-81) obtained from American Type Culture Collection (USA). After 2 h of incubation, the virus/plasma mixture were incubated with Vero cells in 96-well plates (Corning Inc., USA) for 1 h, then replaced with fresh growth medium. The cytopathic effects (CPE) were observed on 5 days post-infection. For each antibody dilution, four duplicate wells were used. Neutralizing antibody titers was calculated by using Reed-Muench method [13] and showed as geometric mean titers (GMTs). The viral genomes reported in this study have been deposited in the Genome Warehouse in the National Genomics Data Center (under project PRJCA003440, publicly accessible at https://bigd.big.ac.cn/gsa). GWHABKH00000001, IPBCAMS-WH-V03 and GWHAORV01000000, IPBCAMS_BJ95.

**1.8 Neutralization of live SARS-CoV-2 by sera/plasma of re-infected patients.** The neutralizing titers were compared with those of 454 convalescent samples from 178 COVID-19 confirmed patients that have been published before [13]. The median neutralizing titer of the cohort was 1:19 (QRI: 1:10-1:28.2; Fig. S4). In particular, the neutralizing ID50s of convalescent samples from case P1 and P3 against D614G variants were1:18.6 and >1:64, respectively (Fig. 1C, Table S3), exceeding 44.3% and 97.4% samples of COVID-19 patients (Fig. S4). Therefore, COVID-19 reinfection could still occur in the presence of measurable levels of neutralizing antibodies.

**1.9 Data availability.** The SARS-CoV-2 genome sequencing data are available at the National Genomics Data Center (accession number PRJCA002533 at https://bigd.big.ac.cn/gsa). Genome consensus sequences are available at Genome Warehouse (accession number GWHAORB01000000 at https://bigd.big.ac.cn/gwh).

**1.10 Code availability.** No unique pipelines or source code were developed for this project.

**Reference**

1. Du P., Ding N., Li J., et al. Genomic surveillance of COVID-19 cases in Beijing. *Nat Commun,* 2020;**11**:5503.

2. National Health Commission of the People's Republic of China, National Administration of Traditional Chinese Medicine. The protocol of diagnosis and treatment of novel coronavirus pneumonia (7th edition). Mar. 3, 2020.

3. Merheb M., Matar R., Hodeify R., et al. Mitochondrial DNA, a powerful tool to decipher ancient human civilization from domestication to music, and to uncover historical murder cases. *Cells,* 2019;**8**.

4. Gill P., Ivanov P.L., Kimpton C., et al. Identification of the remains of the romanov family by DNA analysis. *Nature Genetics,* 1994;**6**:130-135.

5. Chen L., Wang J., Tan L., et al. Highly accurate mtGenome haplotypes from long-read SMRT sequencing can distinguish between monozygotic twins. *Forensic Sci Int Genet,* 2020;**47**:102306.

6. Dobin A., Davis C.A., Schlesinger F., et al. STAR: ultrafast universal RNA-seq aligner. *Bioinformatics,* 2013;**29**:15-21.

7. Martin M. Cutadapt removes adapter sequences from high-throughput sequencing reads. *EMBnet.journal,* 2011;**17**:10.

8. Di L., Fu Y., Sun Y., et al. RNA sequencing by direct tagmentation of RNA/DNA hybrids. *Proc Natl Acad Sci U S A,* 2020;**117**:2886-2893.

9. Langmead B.and Salzberg S.L. Fast gapped-read alignment with Bowtie 2. *Nat Methods,* 2012;**9**:357-9.

10. Langmead B., Trapnell C., Pop M.and Salzberg S.L. Ultrafast and memory-efficient alignment of short DNA sequences to the human genome. *Genome Biol,* 2009;**10**:R25.

11. Li H., Handsaker B., Wysoker A., et al. The Sequence Alignment/Map format and SAMtools. *Bioinformatics,* 2009;**25**:2078-9.

12. Rambaut A., Holmes E.C., O'Toole A., et al. A dynamic nomenclature proposal for SARS-CoV-2 lineages to assist genomic epidemiology. *Nat Microbiol,* 2020;**5**:1403-1407.

13. Ren L., Fan G., Wu W., et al. Antibody responses and clinical outcomes in adults hospitalized with severe COVID-19: a post hoc analysis of LOTUS China trial. *Clin Infect Dis,* 2020.

**Supplemental Figures**


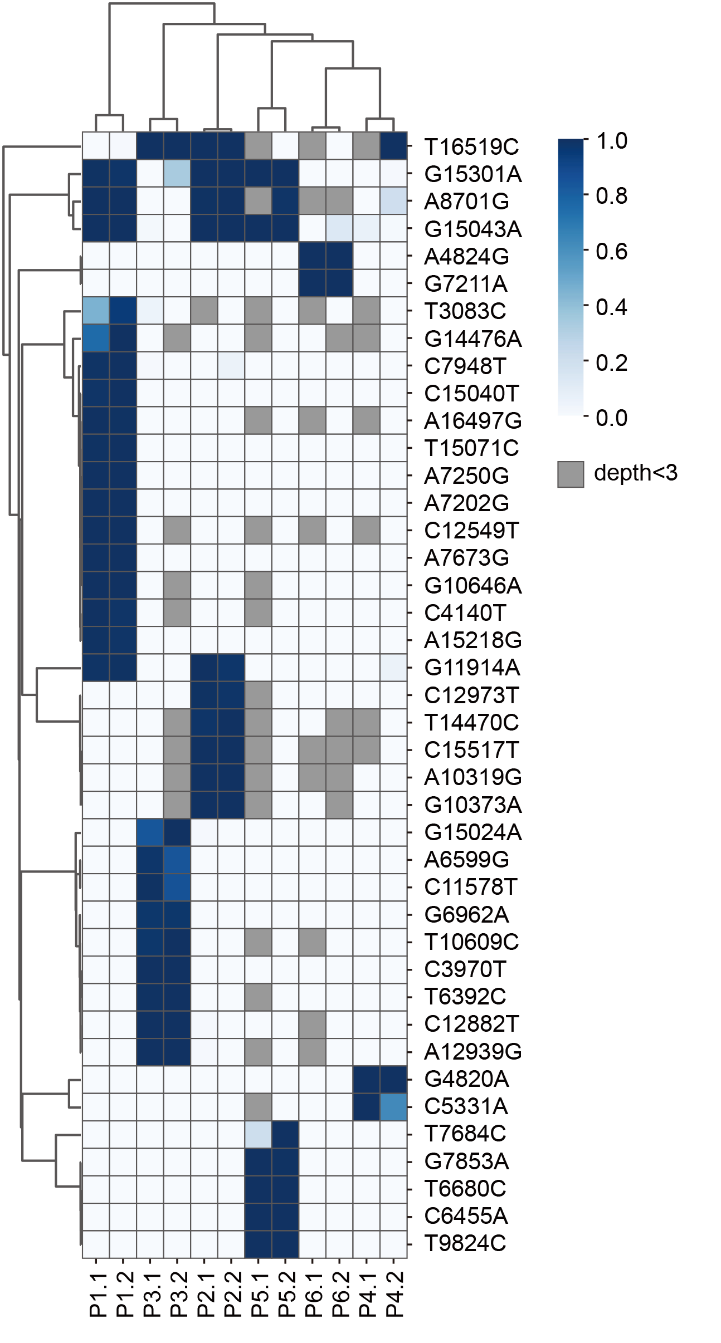


**Supplemental Figure S1. Host mtDNA haplotype analysis for the paired sequencing data of the six re-infected COVID-19 patients.** The heatmap showed the mtDNA SNPs in each sample, with the frequencies represented as the color scale.


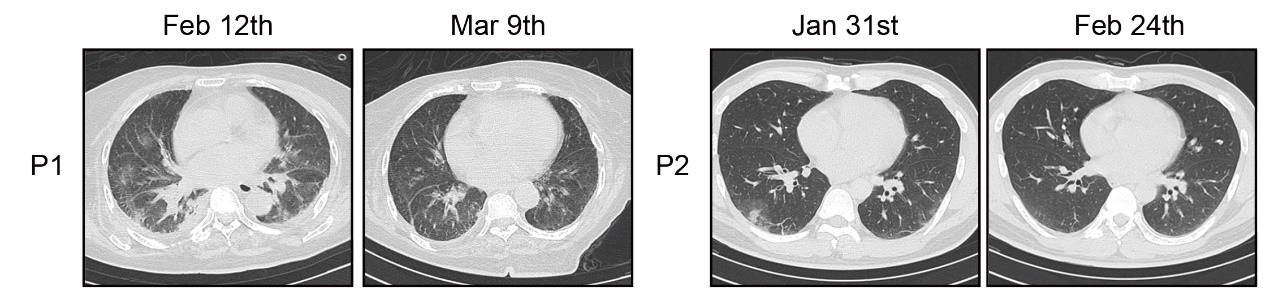


**Supplemental Figure S2. Supplementary high-resolution CT scans of case P1 and P2.** The representative images showed the absorption of inflammatory lesions during the infection intervals of case P1 and P2. P1: February 12^th^ (5 days to onset date, interval period), March 9^th^ (31 days to onset date, interval period); P2: January 31^st^ (7 days to onset date, primary infection period), February 24^th^ (31 days to onset date, interval period).


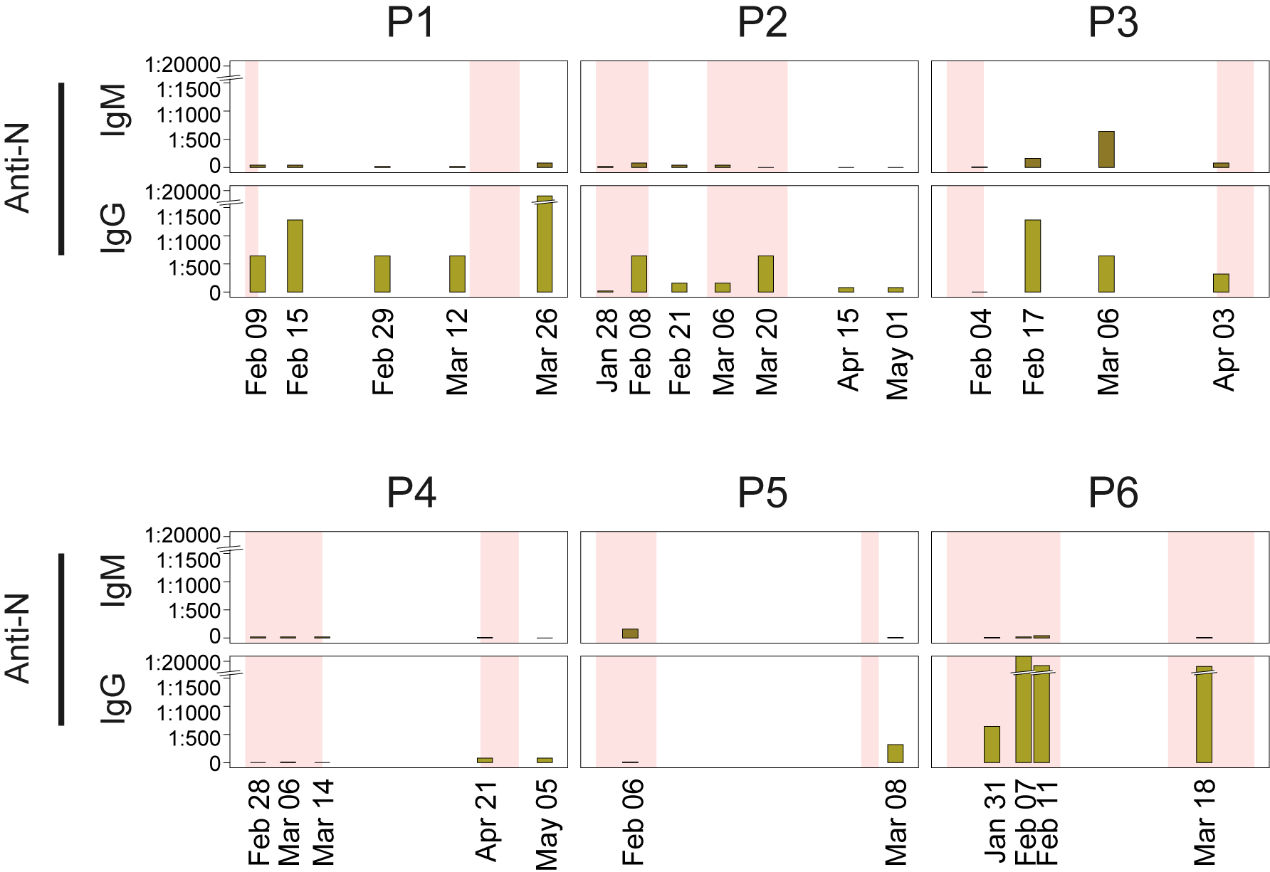


**Supplemental Figure S3. Titer dynamics of antibodies against SARS-CoV-2 NP protein on six patients.** Tan bars, anti-NP IgM titers; khaki bars, anti-NP IgG titers. Background colors represented the primary and secondary infections.


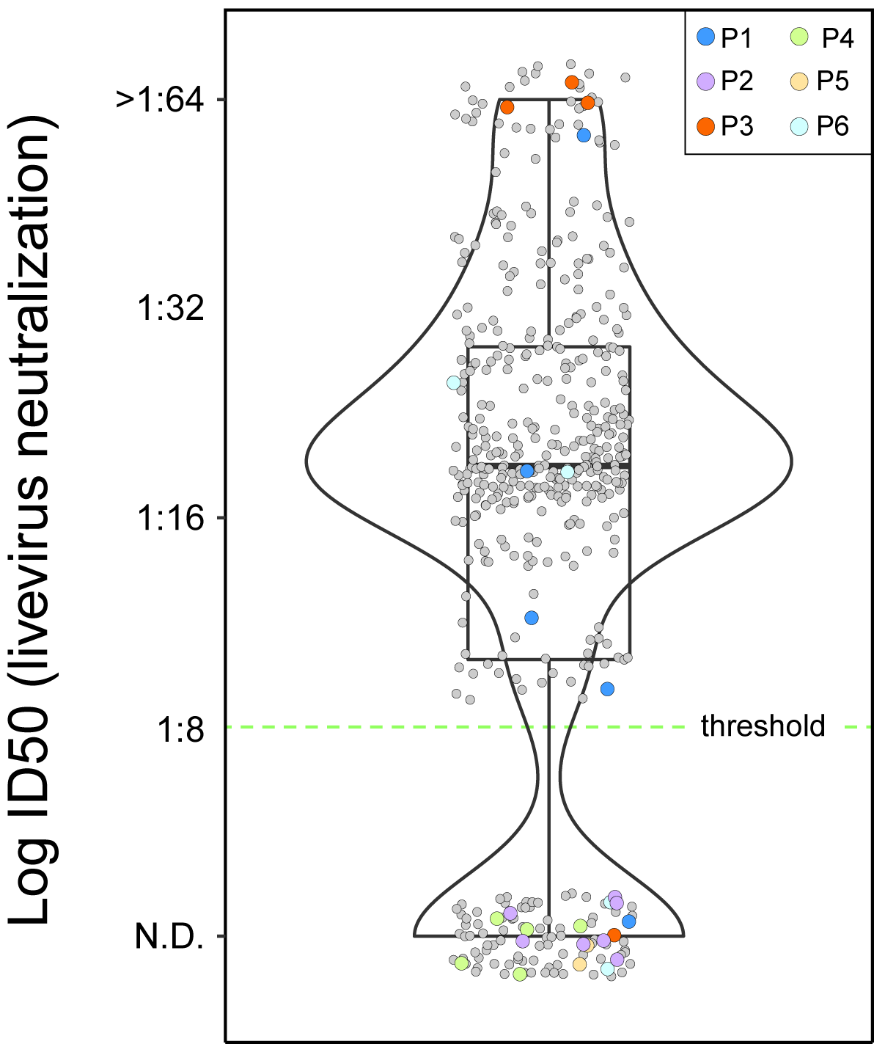


**Supplemental Figure S4.** **Relative neutralization capacity against authentic SARS-CoV-2 virus of re-infected cases.** The re-infected patients' neutralization titers against SARS-CoV-2 virus (reference strain for case P6, D614G variants for case P1-P5) were highlighted in colors, while the published neutralization titers from the other convalescent patients were indicated with white dots. The 25%, 50%, 75% quartiles of observations were plot as boxplot. The horizontal green line indicated the threshold of reported positive results, and values below threshold were designated N.D (not detected).

**Supplemental Table S1. Summary of clinical features and SARS-CoV-2 RNA tests of the six reinfected cases.**

|  | | **P1** | **P2** | **P3** | **P4** | **P5** | **P6** |
| --- | --- | --- | --- | --- | --- | --- | --- |
| **Sex** | | F | M | M | M | F | M |
| **Age** | | 84 | 33 | 59 | 33 | 2 | 74 |
| **Occupation** |  | Retired | Office staff in community government | Retired | Cleaner in an office building | - | Retired |
| **Classification (WHO)** | | Critical | Moderate | Moderate | Moderate | Moderate | Critical |
| **Coexisting chronic illness** | | hypertension, valvular heart disease, cardiorenal syndrome | None | None | None | None | hypertension, coronary heart disease |
| **Interval between two infection episodes (days)** | | 33 | 19 | 57 | 35 | 22 | 24 |
| **Consecutive SARS-CoV-2 negative tests (times)** | | 12 | 5 | 3 | 3 | 3 | 2 |
| **Discharged between two infection stages?** | | No | Yes | Yes | Yes | Yes | Yes |
| **Symptoms** | **Primary infection** | fatigue, cough, expectoration, shortness of breath | fever | fatigue, cough, sore throat, stuffy nose | fever | fever, cough | fever, fatigue, cough, expectoration, dyspnea |
|  | **Secondary infection** | fever | fever, cough, expectoration, shortness of breath | cough, expectoration, stuffy nose | none | none | none |
| **Viral RNA positive duration (Days)** | **Primary infection** | 2 | 18 | 9 | 18 | 8 | 25 |
|  | **Secondary infection** | 9 | 26 | 9 | 9 | 3 | 19 |
| **SARS-CoV-2 positive tests (times)** | **Primary infection** | 3 | 7 | 3 | 7 | 4 | 6 |
|  | **Secondary infection** | 4 | 14 | 3 | 4 | 2 | 7 |
| **Lowest Ct value** | **Primary infection** | 33 | 32 | 29 | 29 | 33 | 33 |
|  | **Secondary infection** | 28 | 28 | 25 | 32 | 37 | 24 |
| **Hospitalization duration (days)** | **Primary infection** | 88 | 30 | 20 | 21 | 16 | 26 |
|  | **Secondary infection** |  | 28 | 12 | 16 | 7 | 23 |
| **Usage of antibiotics** | **Primary infection** | MXF, SUL, CFP, MEM | NO | NO | NO | NO | MXF |
|  | **Secondary infection** | MEM, VAN, FLC | MXF | NO | NO | NO | NO |
| **Usage of antiviral agents** | **Primary infection** | NO | IFN, LPV/r | NO | IFN, LPV/r | IFN | IFN |
|  | **Secondary infection** | NO | RBV | IFN | IFN, LPV/r | IFN | NO |
| **Usage of immuno-suppressants** | **Primary infection** | NO | NO | NO | NO | NO | NO |
|  | **Secondary infection** | NO | NO | NO | NO | NO | NO |
| **Respiratory support** | **Primary infection** | CPAP | NO | NO | NO | NO | Oxygen mask (low-flow) |
|  | **Secondary infection** | Oxygen mask (high-flow) | NO | NO | NO | NO | NO |
| **Dialysis** | **Primary infection** | CRRT | NO | NO | NO | NO | NO |
|  | **Secondary infection** | NO | NO | NO | NO | NO | NO |

MXF: moxifloxacin; SUL: sulbactam; CFP, cefoperazone; MEM: meropenem; VAN: vancomycin; FLC: fluconazole; IFN: interferon; LPV/r: lopinavir-ritonavir; RBV: ribavirin; CQ: chloroquine; CPAP: continuous positive airway pressure; CRRT: continuous renal replacement therapy.

**Supplemental Table S2. Samples sequenced in this study**

| **Sample ID** | **Patient ID** | **Specimen Type** | **Sampling Date** | **Viral Lineage** | **Number of Raw Reads** | **Number of SARS-CoV-2 Reads** | **Ratio (Viral %)** | **Genome Coverage (bp)** | **Genome Coverage (%)** | **Usage** | **Note** |
| --- | --- | --- | --- | --- | --- | --- | --- | --- | --- | --- | --- |
| P1.1 | P1 | Pharyngeal swab | 2020-02-09 | B.2 | 63,305,808 | 58,613,677 | 92.6% | 29872 | 99.9% | confirming reinfection |  |
| P1.2 | P1 | Pharyngeal swab | 2020-03-18 | B.1 | 68,147,274 | 20,105,372 | 29.5% | 26651 | 89.1% | confirming reinfection |  |
| P2.1 | P2 | Sputum | 2020-01-25 | B | 146,373,482 | 132,542,558 | 90.6% | 29890 | 100.0% | confirming reinfection |  |
| P2.2 | P2 | Sputum | 2020-03-26 | B.1.1 | 66,698,666 | 19,356,043 | 29.0% | 29173 | 97.6% | confirming reinfection |  |
| P3.1 | P3 | Sputum | 2020-02-05 | B.2 | 244,694,344 | 128,527,280 | 52.5% | 29838 | 99.8% | confirming reinfection |  |
| P3.2 | P3 | Feces | 2020-04-11 | B.1 | 85,419,986 | 22,465,277 | 26.3% | 29670 | 99.2% | confirming reinfection |  |
| P4.1 | P4 | Sputum | 2020-02-27 | B | 64,491,370 | 20,557,136 | 31.9% | 29879 | 99.9% | confirming reinfection |  |
| P4.2 | P4 | Pharyngeal swab | 2020-04-20 | B.1 | 54,862,226 | 51,167,021 | 93.3% | 23724 | 79.3% | confirming reinfection |  |
| P5.1 | P5 | Pharyngeal swab | 2020-02-06 | B | 254,459,314 | 51,879,708 | 20.4% | 29841 | 99.8% | confirming reinfection |  |
| P5.2 | P5 | Pharyngeal swab | 2020-03-06 | B.1 | 44,786,286 | 167,208 | 0.4% | 28873 | 96.6% | confirming reinfection |  |
| P6.1 | P6 | Pharyngeal swab | 2020-01-31 | A | 35,601,376 | 1,894,230 | 5.3% | 29826 | 99.7% | confirming reinfection |  |
| P6.2 | P6 | Pharyngeal swab | 2020-03-18 | B | 28,672,202 | 7,540,551 | 26.3% | 29378 | 98.2% | confirming reinfection |  |
| P3.2R | P3 | Sputum | 2020-04-11 | B.1 | 67,254,644 | 2,400,060 | 3.6% | 27487 | 91.9% | excluding mix-up |  |
| CP1 |  | Sputum | 2020-03-15 | B.1 | 8,332,860 | 1,638,430 | 19.7% | 29875 | 99.9% | contact tracing | from a close-contact with P1 |
| CP2 |  | Pharyngeal swab | 2020-03-02 | B.1 | 77,996,782 | 72,321,416 | 92.7% | 29556 | 98.8% | contact tracing | from a close-contact with P1 |
| CP3 |  | Sputum | 2020-03-06 | B.1 | 228,174,508 | 223,612,389 | 98.0% | 29873 | 99.9% | contact tracing | from a close-contact with P1 |
| CP4 |  | Sputum | 2020-03-06 | B.1 | 145,780,486 | 86,232 | 0.1% | 22227 | 74.3% | contact tracing | from a close-contact with P5 |

**Supplemental Table S3. Sera/plasma antibody titers and neutralizing capacities against SARS-CoV-2 of re-infected patients**

| **Patient ID** | **Sampling date** | **Sample type** | **Antibodies against SARS-CoV-2** | | | |  | **Neutralization Activity (ID50)** | |
| --- | --- | --- | --- | --- | --- | --- | --- | --- | --- |
|  |  |  | **Anti-RBD IgM (titers)** | **Anti-RBD IgG (titers)** | **Anti-NP IgM (titers)** | **Anti-NP IgG (titers)** |  | **Reference strain** | **D614G variant** |
| P1 | 2020-02-09 | Plasma | 1:20 | 1:20 | 1:40 | 1:640 |  | N.D. | N.D. |
|  | 2020-02-15 | Plasma | 1:80 | 1:40 | 1:40 | 1:1280 |  | 1:10 | 1:8 |
|  | 2020-02-29 | Plasma | 1:40 | 1:320 | 1:20 | 1:640 |  | 1:20 | 1:12.6 |
|  | 2020-03-12 | Plasma | 1:10 | 1:80 | 1:20 | 1:640 |  | 1:13.5 | 1:18.6 |
|  | 2020-03-26 | Plasma | 1:2560 | 1:80 | 1:80 | 1:2560 |  | 1:53.8 | > 1:64 |
| P2 | 2020-01-28 | Serum | N.D. | 1:10 | 1:20 | 1:20 |  | N.D. | N.D. |
|  | 2020-02-08 | Serum | 1:20 | 1:20 | 1:80 | 1:640 |  | N.D. | N.D. |
|  | 2020-02-21 | Serum | 1:40 | 1:40 | 1:40 | 1:160 |  | N.D. | N.D. |
|  | 2020-03-06 | Serum | N.D. | N.D. | 1:40 | 1:160 |  | N.D. | N.D. |
|  | 2020-03-20 | Serum | 1:10 | N.D. | N.D. | 1:640 |  | N.D. | N.D. |
|  | 2020-04-15 | Serum | N.D. | N.D. | N.D. | 1:80 |  | N.D. | N.D. |
|  | 2020-05-01 | Serum | N.D. | N.D. | N.D. | 1:80 |  | N.D. | N.D. |
| P3 | 2020-02-04 | Serum | 1:40 | N.D. | 1:10 | N.D. |  | N.D. | N.D. |
|  | 2020-02-17 | Serum | 1:320 | 1:20 | 1:160 | 1:1280 |  | 1:38.8 | > 1:64 |
|  | 2020-03-06 | Serum | 1:160 | 1:160 | 1:640 | 1:640 |  | > 1:64 | > 1:64 |
|  | 2020-04-03 | Serum | 1:160 | 1:40 | 1:80 | 1:320 |  | 1:45.5 | > 1:64 |
| P4 | 2020-02-28 | Serum | N.D. | N.D. | 1:20 | N.D. |  | N.D. | N.D. |
|  | 2020-03-06 | Serum | 1:40 | N.D. | 1:20 | 1:10 |  | N.D. | N.D. |
|  | 2020-03-14 | Serum | 1:40 | N.D. | 1:20 | N.D. |  | N.D. | N.D. |
|  | 2020-04-21 | Serum | 1:10 | N.D. | 1:10 | 1:80 |  | N.D. | N.D. |
|  | 2020-05-05 | Serum | N.D. | N.D. | N.D. | 1:80 |  | N.D. | N.D. |
| P5 | 2020-02-06 | Serum | 1:20 | N.D. | 1:160 | 1:10 |  | N.D. | N.D. |
|  | 2020-03-08 | Serum | 1:10 | 1:320 | 1:10 | 1:320 |  | N.D. | N.D. |
| P6 | 2020-01-31 | Plasma | 1:10 | N.D. | 1:10 | 1:640 |  | N.D. | N.D. |
|  | 2020-02-07 | Plasma | 1:40 | 1:160 | 1:20 | 1:20480 |  | N.D. | N.D. |
|  | 2020-02-11 | Plasma | 1:320 | 1:320 | 1:40 | 1:10240 |  | 1:18.5 | 1:16 |
|  | 2020-03-18 | Plasma | 1:80 | 1:160 | 1:10 | 1:2560 |  | 1:22.4 | 1:22.4 |

N.D., not detected (< 1:10 for antibody detection; < 1:100 for neutralization activity against pseudoviral particle); < 1:8 for neutralization activity against live virus)

**Supplemental Table S4. Lymphocyte count and percentage in the secondary infections and infection intervals.**

| **Patient** | **Date** | **Episode** | **Count (****x 10^9^/L) ^*^** | **Ratio (%) ^#^** |
| --- | --- | --- | --- | --- |
| P1 | 2020/2/8 | Primary infection | 1.36 | 19.72 |
|  | 2020/2/13 | Interval | 1.57 | 10.24 |
|  | 2020/2/19 | Interval | 2.04 | 20.64 |
|  | 2020/2/25 | Interval | 1.59 | 16.72 |
|  | 2020/3/2 | Interval | 0.88 | 22.24 |
|  | 2020/3/8 | Interval | 0.88 | 17.52 |
|  | 2020/3/17 | Secondary infection | 1.39 | 20.7 |
|  | 2020/3/20 | Secondary infection | 0.64 | 15.22 |
| P2 | 2020/1/25 | Primary infection | 1.09 | 14.02 |
|  | 2020/2/5 | Primary infection | 1.51 | 36.7 |
|  | 2020/2/12 | Interval | 1.77 | 40.2 |
|  | 2020/2/20 | Interval | 1.68 | 36 |
|  | 2020/3/2 | Secondary infection | 1.01 | 8.12 |
|  | 2020/3/9 | Secondary infection | 1.36 | 24.8 |
|  | 2020/3/20 | Secondary infection | 1.47 | 34.4 |
| P3 | 2020/2/4 | Primary infection | 1.04 | 31 |
|  | 2020/2/17 | Interval | 1.35 | 34.6 |
|  | 2020/3/6 | Interval | 1.31 | 36.1 |
|  | 2020/4/7 | Secondary infection | 1.5 | 39.4 |
| P4 | 2020/2/28 | Primary infection | 2.23 | 34.4 |
|  | 2020/3/14 | Primary infection | 1.63 | 18.42 |
|  | 2020/4/20 | Secondary infection | 1.48 | 27.7 |
|  | 2020/4/21 | Secondary infection | 1.54 | 31.3 |
| P5 | 2020/2/3 | Primary infection | 5.45 | 55.11 |
|  | 2020/3/4 | Secondary infection | 7.12 | 71.31 |
| P6 | 2020/2/2 | Primary infection | 0.78 | 14.72 |
|  | 2020/2/12 | Primary infection | 1.57 | 23.8 |
|  | 2020/3/11 | Secondary infection | 1.63 | 38.1 |
|  | 2020/3/18 | Secondary infection | 2.09 | 40 |
|  | 2020/3/27 | Secondary infection | 1.78 | 40 |

* Reference range: 1~5 x 10^9^/L; # Reference range: 20~40%.

**Supplemental Table S5. T cell counts of the re-infected patients**

| **Patient ID** | **Sampling date** | **Episode** | **CD4^+^T lymphocyte (cells/ml)** | |  | **CD8^+^T lymphocyte (cells/ml)** | |
| --- | --- | --- | --- | --- | --- | --- | --- |
|  |  |  | **Value** | **Reference range** |  | **Value** | **Reference range** |
| P1 | 2020/2/9 | Primary infection | 448 | 706-1125 |  | 291 | 320-1250 |
|  | 2020/3/21 | Secondary infection | 201 |  |  | 209 |  |
|  | 2020/3/29 | Convalescence | 342 |  |  | 332 |  |
|  | 2020/4/9 | Convalescence | 322 |  |  | 151 |  |
| P2 | 2020/3/2 | Secondary infection | 420 | 706-1125 |  | 434 | 320-1250 |
|  | 2020/3/8 | Secondary infection | 659 |  |  | 531 |  |
| P3 | 2020/2/4 | Primary infection | 372 | 706-1125 |  | 289 | 320-1250 |
|  | 2020/2/17 | Interval | 518 |  |  | 407 |  |
|  | 2020/4/7 | Secondary infection | 557 |  |  | 486 |  |
|  | 2020/4/13 | Convalescence | 427 |  |  | 373 |  |
| P4 | 2020/2/27 | Primary infection | 491 | 706-1125 |  | 1229 | 320-1250 |
|  | 2020/3/4 | Primary infection | 381 |  |  | 1157 |  |
|  | 2020/3/16 | Interval | 443 |  |  | 823 |  |
| P6 | 2020/2/2 | Primary infection | 318 | 706-1125 |  | 49 | 320-1250 |
|  | 2020/2/13 | Primary infection | 703 |  |  | 148 |  |
